# An ex vivo permissivity assay to assess replication of the oncolytic virus VSV-GP in patient-derived tumor samples

**DOI:** 10.1038/s41388-026-03822-9

**Published:** 2026-05-26

**Authors:** Benjamin Schoeps, Stefanie Estermann, Susanne Berchtold, Julia Beil, Can Yurttas, Melissa Mayr, André Volland, Aida Guerrero Tort, Marlies Christina Glatz, Fabian Martin, Irina Smirnow, Nicole Klammsteiner, Linus D. Kloker, Michaela Smolle, Tobias Nolden, Regina Rettenmaier, Rainer Kleemann, Theresa Schwaiger, Monika Petersson, Knut Elbers, Christiane Knobbe-Thomsen, Ulrich M. Lauer, Krishna Das

**Affiliations:** 1 ViraTherapeutics GmbH, Rum, Austria; 2 Department of Medical Oncology and Pneumology, Virotherapy Center Tübingen (VCT), Medical University Hospital, Tübingen, Germany; 3 German Cancer Consortium (DKTK), partner site Tuebingen, a partnership between DKFZ and University Hospital Tuebingen, Tuebingen, Germany; 4 Department of General, Visceral and Transplant Surgery, University Hospital of Tübingen, Tübingen, Germany; 5 Boehringer Ingelheim Pharma GmbH & Co. KG, Ingelheim, Germany

**Keywords:** Cancer microenvironment, Cancer models, Cancer immunotherapy, Immunotherapy, Gene therapy

## Abstract

The sparking interest in oncolytic viruses (OV) faces challenges in clinical translation due to limitations of pre-clinical models and the lack of predictive biomarkers for OV activity. Furthermore, functional assays which could determine permissivity of human tumors to OVs in a straightforward way are still lacking. Here, we present a novel ex vivo permissivity assay to precisely quantify replication of OVs in viable patient-derived tumors. As example, replication of reporter protein-expressing oncolytic VSV-GP variants was tracked via fluorescence, luminescence or qPCR across 133 patient-derived tumor samples (resection fragments/slices, biopsies) spanning more than 20 tumor entities. Based on the results of our comprehensive testing and by employing VSV-GP-NanoLuc-Katushka, we were able to establish a semi-automated permissivity assay with minimal hands-on time, allowing robust and real-time tracking of viral replication in patient-derived tumor samples. Given the urgent demand for innovative cancer treatments, this novel permissivity assay could be applied to select patients with tumors permissive to OV replication and might thus also show an enhanced clinical response.

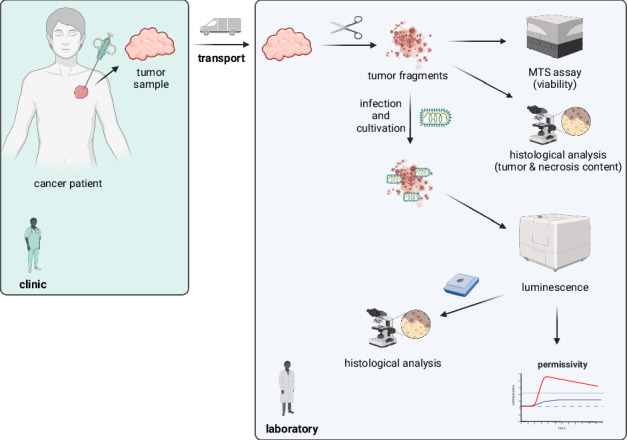

## Introduction

Oncolytic viruses (OVs) have gained increasing attention in recent years [1, 2], especially due to their high potential in combination with other immunotherapies like checkpoint inhibitors [3–5]. Upon infection of their cancerous target cells, OVs replicate, trigger tumor cell lysis and can ultimately lead to inflammation in the tumor microenvironment, including recruitment and activation of immune cells, as well as an immune response against tumor antigens released from lysed cells [6]. One promising OV candidate is vesicular stomatitis virus (VSV), a bullet-shaped negative-stranded RNA virus with a genome encoding five major proteins: nucleoprotein (N), phosphoprotein (P), matrix protein (M), glycoprotein (G), and viral polymerase (L) [7]. During replication, VSV transcribes its genes sequentially, starting from the 3′ genome end, resulting in a natural gradient of transcript abundance with N expressed earliest and at highest levels and L expressed latest and at lowest levels [7]. VSV was recently pseudotyped with the envelope glycoprotein (GP) of the lymphocytic choriomeningitis virus (LCMV) to reduce neurotoxicity [8]. VSV-GP is currently tested in a phase I study in advanced and refractory solid tumors (NCT05155332) [3]. Although more than 97 clinical studies with OVs have been undertaken between 2000 and 2020 [9], translation of promising pre-clinical findings into the clinic remains challenging, and talimogene laherparepvec (T-VEC) still is the only widely approved OV therapeutic concept so far [1]. The complex mode of action of OVs and the lack of predictive (i) biomarkers [1, 10] and/or (ii) functional assays significantly contribute to this low clinical success rate. The recent failure of an unstratified phase III clinical trial (MASTERKEY-265) investigating the effect of T-VEC plus pembrolizumab in advanced melanomas [11] further highlights the urgent demand for patient stratification in OV therapies. This need is equally evident in the recurrent‑disease setting, as demonstrated by the MASTERKEY‑115 study, which reported markedly different response rates depending on the pattern of resistance to prior PD‑1 therapy [12].

The high need of biomarkers for OV activity also triggered a recent study to screen patient-derived pancreatic tumor samples for biomarkers of efficacy of various OVs [13]. However, the authors concluded that instead of testing for single biomarkers, ex vivo sensitivity and transcriptome analysis should be employed for patient stratification [13]. Currently, a straightforward and reproducible assay to assess ex vivo permissivity - namely, the intrinsic capacity of tumors to support viral replication - has not been established. Such a permissivity assay has to meet several prerequisites. First, it must be applicable to patient-derived tumor material, since animal and in vitro model systems do not adequately mirror patient tumors [1, 14] and mouse models have only a limited predictive value for the response to OV therapy in patients [14]. Second, the assay should retain the tumor ecosystem in its native structure, including cancer cells and the tumor microenvironment (TME), since not only molecular alterations in tumor cells but also components of the TME determine permissivity to OVs [10]. Third, it must be applicable to a variety of tumor entities. Finally, the assay should be reproducible and simple enough to be implemented in a variety of laboratories, as access to viable tumor tissue is restricted to specific locations, and proximity to clinical sites is crucial for enabling timely testing.

Here, we established a novel permissivity assay to detect OV replication in patient-derived tumor samples. For this, we exemplarily employed reporter protein expressing variants of VSV-GP, which is currently tested in phase I clinical trials, and compared various available patient-derived ex vivo tumor model systems, also termed patient tumor avatars [15, 16], to assess suitability for permissivity testing. These tumor models encompass tumor slices [17], tumor biopsies [18] and tumor fragments [19]. This novel permissivity assay might be used as a predictive companion diagnostic positively selecting for cancer patients who not only are permissive to OV replication but might also show an enhanced clinical response.

## Materials and methods

### Human biospecimen acquisition

Tumor samples were obtained by BioIVT or the Medical University Hospital Tübingen. All procedures were conducted in accordance with the declaration of Helsinki and were approved by the IRB of the University of Tübingen (ethics committee of the Faculty of Medicine of University of Tübingen, reference no. 151/2022BO2) or the local governmental institution (for BioIVT samples). Informed consent was obtained from all individuals involved in the study. Patients with medically indicated tumor resections or biopsies were recruited through the local tumor boards and fresh tumor tissue not required for regular pathological workup was processed as described below. An overview on the employed tumor samples is shown in Table 1.

**Table 1:** Overview on the employed human tumor samples.

Patient ID	Tumor entity	Fluorescence	qPCR	Luminescence
Patient #086	Bladder Carcinoma	x	x	x
Patient #091	Bladder Carcinoma	x	x	x
Patient #100	Bladder Carcinoma	x	x	x
Patient #108	Bladder Carcinoma	x		x
Patient #009	Bladder Carcinoma	x	x	
Patient #033	Bladder Carcinoma	x	x	
Patient #041	Bladder Carcinoma	x	x	
Patient #113	Bladder Carcinoma	x	x	x
Patient #116	Bladder Carcinoma	x	x	x
Patient #118	Bladder Carcinoma	x	x	x
Patient #084	Breast Carcinoma	x	x	x
Patient #080	Cecum Carcinoma	x	x	
Patient #017	Cholangiocellular Carcinoma	x	x	
Patient #036	Cholangiocellular Carcinoma	x	x	
Patient #049	Cholangiocellular Carcinoma	x	x	
Patient #054	Cholangiocellular Carcinoma	x	x	
Patient #110	Cholangiocellular Carcinoma	x	x	x
Patient #181	Cholangiocellular Carcinoma	x		x
Patient #039	Colon Carcinoma	x	x	
Patient #062	Colon Carcinoma	x		
Patient #093	Colon Carcinoma	x	x	x
Patient #104	Colon Carcinoma metastasis	x	x	x
Patient #180	Colon Carcinoma metastasis	x		x
Patient #013	Colon Carcinoma metastasis	x	x	
Patient #046	Colon Carcinoma metastasis	x	x	
Patient #012	DSRCT	x	x	
Patient #071	DSRCT	x	x	
Patient #175	DSRCT metastasis	x		x
Patient #076	Duodenum Carcinoma	x	x	
Patient #164	Gastric Carcinoma	x		x
Patient #176	Gastric Carcinoma	x		x
Patient #178	Gastric Carcinoma	x		x
Patient #043	Gastric Carcinoma	x	x	
Patient #141	Gastric Carcinoma	x		x
Patient #056	Gastric Carcinoma	x	x	
Patient #132	Gastric Carcinoma	x		x
Patient #089	Gastric Carcinoma	x	x	x
Patient #021	Gastric Carcinoma metastasis	x	x	
Patient #024	Gastric Carcinoma metastasis	x	x	
Patient #088	GIST	x	x	x
Patient #092	GIST	x	x	x
Patient #115	GIST metastasis	x	x	x
Patient #139	Glioblastoma	x		x
Patient #142	Glioblastoma	x		x
Patient #097	Ileum Carcinoma	x	x	x
Patient #099	Jejunum Carcinoma	x	x	x
Patient #103	Kidney Carcinoma	x	x	x
Patient #059	Kidney Carcinoma	x	x	
Patient #161	Kidney Carcinoma	x		x
Patient #032	Kidney Carcinoma	x	x	
Patient #129	Kidney Carcinoma	x		x
Patient #061	Kidney Carcinoma	x	x	
Patient #179	Kidney Carcinoma	x		x
Patient #075	Kidney Carcinoma	x	x	
Patient #083	Kidney Carcinoma	x	x	x
Patient #064	Kidney Carcinoma	x	x	
Patient #038	Kidney Carcinoma	x	x	
Patient #040	Kidney Carcinoma	x	x	
Patient #044	Kidney Carcinoma	x	x	
Patient #055	Kidney Carcinoma	x	x	
Patient #060	Kidney Carcinoma	x	x	
Patient #070	Kidney Carcinoma	x	x	
Patient #077	Kidney Carcinoma	x	x	
Patient #095	Kidney Carcinoma	x	x	x
Patient #096	Kidney Carcinoma	x	x	x
Patient #121	LAMN	x		x
Patient #123	LAMN	x		
Patient #008	Liposarcoma	x	x	
Patient #050	Liposarcoma	x	x	
Patient #102	Liposarcoma	x	x	x
Patient #107	Liposarcoma	x	x	x
Patient #134	Liposarcoma	x		x
Patient #114	Liver Carcinoma	x	x	x
Patient #119	Liver Carcinoma	x	x	x
Patient #120	Liver Carcinoma	x	x	
Patient #124	Liver Carcinoma	x		x
Patient #127	Liver Carcinoma	x		x
Patient #138	Liver Carcinoma	x		x
Patient #144	Liver Carcinoma	x		x
Patient #156	Liver Carcinoma	x		x
Patient #160	Liver Carcinoma	x		x
Patient #167	Lung Carcinoma	x		x
Patient #001	Lung Carcinoma	x	x	
Patient #019	Lung Carcinoma	x	x	
Patient #069	Lung Carcinoma	x	x	
Patient #073	Lung Carcinoma	x	x	
Patient #105	Lung Carcinoma	x	x	x
Patient #106	Lung Carcinoma	x	x	x
Patient #125	Lung Carcinoma	x		x
Patient #133	Lung Carcinoma	x		x
Patient #168	Lung Carcinoma	x		x
Patient #052	Lung Carcinoma	x	x	
Patient #057	Lung Carcinoma	x	x	
Patient #085	Lung Carcinoma	x	x	x
Patient #087	Lung Carcinoma	x	x	x
Patient #152	Lung Carcinoma	x	x	x
Patient #143	Lung Carcinoma	x		x
Patient #145	Lung Carcinoma	x		x
Patient #147	Lung Carcinoma	x		x
Patient #148	Lung Carcinoma	x		x
Patient #150	Lung Carcinoma	x		x
Patient #153	Lung Carcinoma	x		x
Patient #158	Lung Carcinoma	x		x
Patient #163	Lung Carcinoma	x		x
Patient #165	Lung Carcinoma	x		x
Patient #007	Lung Carcinoma	x	x	
Patient #015	Lung Carcinoma	x	x	
Patient #016	Lymphnode Carcinoma	x	x	
Patient #022	Lymphnode Carcinoma	x	x	
Patient #023	Lymphnode Carcinoma	x	x	
Patient #098	Melanoma	x	x	x
Patient #122	Melanoma	x		x
Patient #126	Melanoma	x		x
Patient #136	Melanoma	x		x
Patient #157	Melanoma	x		x
Patient #162	Melanoma	x		x
Patient #174	Melanoma	x		x
Patient #025	Melanoma metastasis	x	x	
Patient #030	Pancreatic Carcinoma	x	x	
Patient #031	Pancreatic Carcinoma	x	x	
Patient #045	Pancreatic Carcinoma	x	x	
Patient #063	Pancreatic Carcinoma	x	x	
Patient #068	Pancreatic Carcinoma	x	x	
Patient #074	Pancreatic Carcinoma	x	x	
Patient #011	Pancreatic Carcinoma	x	x	
Patient #026	Pancreatic Carcinoma	x	x	
Patient #184	Prostate Carcinoma	x		x
Patient #185	Prostate Carcinoma	x		x
Patient #186	Prostate Carcinoma	x		x
Patient #187	Prostate Carcinoma	x		x
Patient #111	Prostate Carcinoma	x	x	x
Patient #117	Prostate Carcinoma	x	x	x
Patient #072	Rectal Carcinoma	x	x	
Patient #082	Rectal Carcinoma	x	x	x
Patient #128	Sarcoma	x		x
Patient #149	Sarcoma metastasis	x		
Patient #051	Sigma Carcinoma	x	x	
Patient #154	Sigma Carcinoma metastasis	x		x
Patient #173	Sigma Carcinoma metastasis	x		x
Patient #014	Thorax Carcinoma	x	x	

### Culture of patient-derived tumor samples

Cultivation of tumor samples was based on a previously described protocol [17]. Following surgical resection, samples were placed in MACS Tissue Storage Solution (Miltenyi Biotec, #130-100-008) supplemented with 1% Penicillin/Streptomycin (ThermoFisher, #15070063) and 1% Amphotericin B (ThermoFisher, #15290026), and transported at 4 °C to the lab. Transport and all processing steps occurred within 24 h post-surgery. Tumor slices were prepared using a LeicaVT1200S vibratome, as described previously [17], and biopsies were obtained employing a Unicore Punch Kit (Qiagen, #WHAWB100073). Both, tumor biopsies and slices, were collected in PBS (Gibco, #10010023) supplemented with 1% Penicillin-Streptomycin (ThermoFisher, #15070063). For tumor fragment generation, a designated tumor piece was weighed and transferred to a 1.5 ml Eppendorf tube. Using sterile scissors, the piece was cut into ~1 mm³ fragments and resuspended in culture media in a volume (µl) adjusted to four times of the tissue weight (mg). For instance, 100 mg of tumor fragments were resuspended in 400 µl of culture media. All tumor samples were cultured in OncoPro Basal Medium supplemented with OncoPro BSA, OncoPro Supplement, B-27 Supplement, Penicillin-Streptomycin (ThermoFisher, #15070063) and Primocin (Invivogen, #ant-pm-05) according to manufacturer’s instructions (ThermoFisher, #A5701201). Tumor slices and biopsies were placed in 96 Well Clear Bottom White Plates (Corning, #165306) containing 100 µl complete medium. Tumor fragments were seeded into 75 µl of complete culture media by adding 25 µl of the generated suspension to each well. The obtained fragments, biopsies or slices of each tumor sample were split regularly into five treatment groups: (i) baseline fixation for histological analysis, (ii) mock control, (iii) UV-inactivated VSV-GP control, (iv) active VSV-GP, and (v) MTS assay. Mock samples received 100 µl culture media only. If not stated otherwise, UV-inactivated VSV-GP and VSV-GP samples were infected with 1 × 10^7^ TCID_50_ infectious virus particles. UV-inactivation was performed employing a Boekel Scientific Crosslinker (#234100) with TIME mode set to 5 min (**≙** 3119 mJ) [17]. Inactivation was validated by qPCR and TCID_50_. After infection, samples were incubated at 37 °C and 5% CO_2_ for 2 h, washed twice with DMEM (Gibco, #21063-029) and covered with 220 µl complete culture media. Two 60 µl supernatant samples (baseline) were collected and stored at −80 °C for subsequent qPCR analysis. Samples were then incubated for 72 h at 37 °C and 5% CO_2_.

For the luminescence-based assay, 1 µl of Endurazine (Promega, #N2571) was added to tumor samples covered with 100 µl complete culture media, and luminescence was recorded starting from 1 h post Endurazine addition (baseline value). In addition, tumor samples were analyzed via fluorescence microscopy every 24 h and scored according to the extent of viral spread. At 72 h post infection (hpi) supernatants were collected for qPCR. For whole-well qPCR of tumor fragments, the respective fragments and their supernatants were transferred to Eppendorf tubes and stored at −80 °C until further processing. All other samples were fixed in 4% formaldehyde for 20 min at room temperature and then stored in PBS until paraffin embedding, hematoxylin and eosin (H&E) staining and histological analyses.

### Murine studies

Animal housing and experimental procedures were conducted according to the French and European Regulations and the National Research Council Guide for the Care and Use of Laboratory Animals. The animal facility is authorized by the French authorities (Dijon: Agreement N° C 21 231 011 EA). All animal procedures (including surgery, anesthesia and euthanasia as applicable) used in the current study were submitted to the Institutional Animal Care and Use Committee of Oncodesign Services (Oncomet) approved by French authorities [CNREEA agreement N° 91 (Oncodesign Services)]. Seven to nine week old female Balb/c mice were subcutaneously implanted with 1 × 10^6^ CT26-CL25-IFNAR^−/−^ cells in the right flank. Mice were housed under BSL2 conditions on a 12 h light/dark cycle with free access to food and water. Tumor size was measured 2–3 times weekly using a caliper, and tumor volume was calculated with the following formula:

$${\rm{Tumor\; volume}}=\frac{{{\rm{width}}}^{2}\times {\rm{length}}}{2}$$

Upon reaching 400 mm^3^, mice were euthanized by CO_2_ asphyxiation followed by cervical dislocation. Tumors were transported over night at 4 °C in MACS Tissue Storage Solution (Miltenyi Biotec, #130-100-008) supplemented with 1% Penicillin/Streptomycin (ThermoFisher, #15070063) and 1% Amphotericin B (ThermoFisher, #15290026), and processed as human tumors, except culture in DMEM (Gibco, #21063-029) supplemented with 1% Penicillin-Streptomycin.

### Assessment of viability and tumor cell content

Viability was assessed at baseline using MTS assay according to manufacturer’s instructions. Briefly, 20 µl MTS reagent was added to 100 µl complete culture media containing tumor slices, fragments or biopsies and incubated for 2 h at 37 °C and 5% CO_2_. Afterwards, absorbance of the supernatant at 490 nm was detected using Tecan Spark 10 M (Tecan). The absorbance value obtained with wells containing media with MTS reagent only (blank) were subtracted from the absorbance values of tumor samples to obtain the corrected absorbance. Some tumor samples were treated with Triton-X (Merck) to determine the background signal of the MTS assay that is obtained with dead samples. For this, tumor fragments or slices were incubated with 1% Triton-X in culture media for 15 min at 37 °C. Afterwards the samples were washed with media and the viability was determined as described above.

Histological analysis of tumor samples was performed at baseline and after 72 h of cultivation. For this, tumor samples were fixed in 4% formaldehyde, paraffin-embedded, cut into 4 µm sections and stained with H&E. Slides were scanned using a 3D Histech Panoramic Scan II at 40x and evaluated within the HALO software environment (Indica Labs). Tumor content was semi-quantitatively graded ranging from 0 = no tumor cells; 1 ≈ 10 tumor cells (e.g., one duct); 2 ≈ 100 tumor cells; 3 ≈ 1000 or more tumor cells. Tumor samples with corrected absorbance below 0.04 (tumor fragments) or 0.052 (tumor slices), and tumor samples with no tumor content or high necrosis content (>80% of necrosis) were excluded from permissivity studies. From 179 collected tumors, a total of 133 passed the quality control.

### Analysis of viral replication via RNAscope

For viral RNA detection – both minus and plus strand – commercially available RNAscope probes were used: LS V-VSV-N probe (ACD, #453008) for the detection of positive-strand genomic and N-mRNA and LS V-VSV-sense for the detection of negative-strand N-region of VSV genomic RNA (ACD, #858988) [20]. The hybridization protocol was carried out using the RNAscope™ 2.5 LS Reagent Kit-BROWN (ACD, # 322100) and the Bond Polymer Refine Detection Kit (Leica, #DS9800) on a fully automated BOND RX stainer (Leica). After cover slipping, slides were scanned as described above.

### Viruses

Recombinant viruses VSV-GP-Katushka and VSV-GP-GFP were produced as described previously [8, 21]. VSV-GP-NanoLuc-Katushka was generated by Gibson cloning of a NanoLuciferase-Katushka cassette (separated through a P2A site) into the 5th position of the viral genome.

### Fluorescence microscopy

A fluorescence-based semi-quantitative scoring system [17] was employed to determine viral replication in tumor slices. Briefly, VSV-GP-Katushka replication was determined by fluorescence microscopy every 24 h and classified into different groups according to the degree of viral replication, ranging from 0 (no Katushka-positive cells), 1 (single positive cells), 2 (several positive cells, few positive patches possible), 3 (large positive clusters), 4 (multiple positive clusters, only few negative cells) to 5 (all cells in tumor slice Katushka-positive). Tumor slices with a score of 3 or more were considered permissive. Fluorescence images were captured on the ZEISS Celldiscoverer 7. Images of each tumor were processed and exported using identical settings in ZEN Blue: tile fusion, z-stack orthogonal projection, and TIFF export.

### RNA isolation and qPCR

Viral RNA levels were determined after wash (baseline) and at 72 hpi in supernatants of tumor slices, and at 72 hpi in entire fragment wells (tissue + supernatant) employing qPCR. For lysis, 100 µl frozen fragment aliquots were thawed and transferred to Lysis tube E (Analytik Jena, #845-CS-1070) containing 200 µl of 3% 2-mercaptoethanol (Merck, #M6250) in Buffer RLT Plus (Qiagen, #1053393). Samples were homogenized using Speed Mill Plus (Analytik Jena, #845-00007) (1 min) and cooled on ice (1 min). This cycle was repeated twice. An additional 300 µl of Buffer RLT Plus was added for dilution. Samples were vortexed, briefly centrifuged, and aliquoted for RNA extraction. RNA from tumor culture lysates and supernatants was extracted employing the MagMAX Viral Nucleic Acid Isolation Kit (ThermoFisher, #A42352). Finally, VSV-N copies were measured using iTaq™ Universal Probes One-Step Kit (BioRad, #1725141) with VSV-N primers: forward: 5′-AGTACCGGAGGATTGACGACTAAT-3′; reverse: 5′-TCAAACCATCCGAGCCATTC-3′ and the probe 5′-ACCGCCACAAGGCAGAGATGTGGT-3′. Amplification protocol: 50 °C for 10 min, 95 °C for 2 min and 40 cycles of 95 °C for 15 s and 60 °C for 30 s. A standard curve was set up by using VSV RNA in a concentration from 10^7^ to 10^1^ copies/ml. All qPCR samples were measured in technical triplicates.

### Detection of luminescence

Luciferase (NanoLuc) activity of tumor samples infected with VSV-GP-NLuc-Kat was analyzed employing the BioSpa-SynergyH1 combination (Agilent) or the Tecan Spark 10 M. The exposure time was set to 1000 ms. The peak value obtained during 72 h of cultivation of each sample was used to assess permissivity and the luminescence value obtained 1 h after addition of Endurazine was employed as baseline luminescence value. If the BioSpa-SynergyH1 combination was used, the measurements were performed every 2 h.

### Data analysis, statistics, and figure preparation

Figures were created employing Adobe Illustrator, BioRender.com and GraphPad Prism 10. Statistical analyses were conducted using GraphPad Prism 10, taking into account data normality and variance. Details regarding sample size, variability, and the specific statistical tests applied are provided in the respective figure legends. Sample sizes were determined by clinical material availability, and no statistical procedures were used to predetermine sample size.

## Results

### Quality control of patient-derived tumor samples

**Fig. 1:**
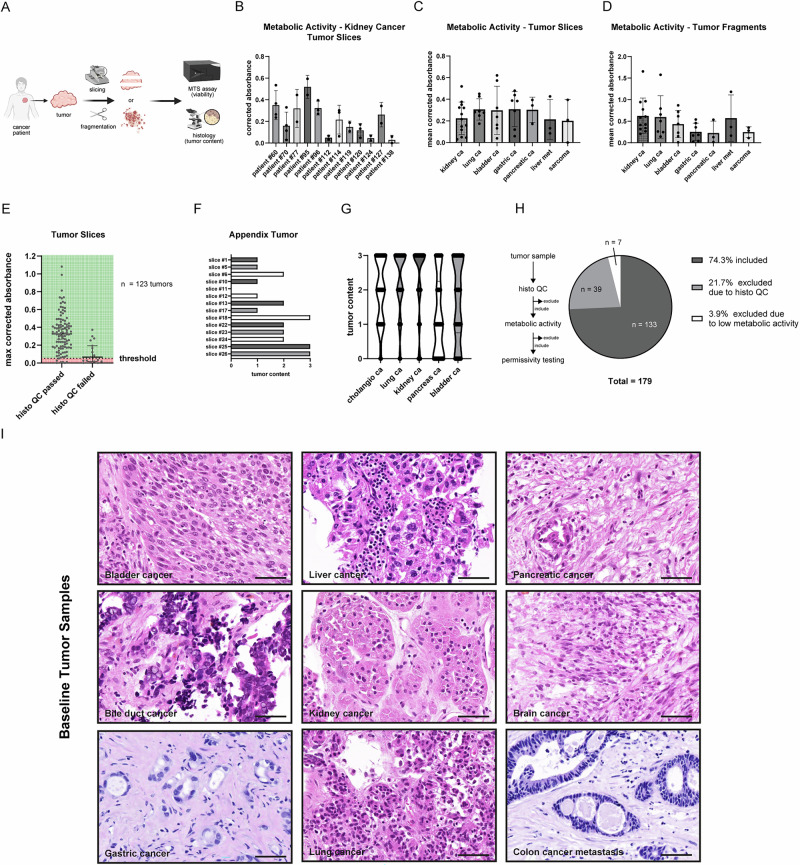
Quality control of patient-derived tumor samples. **A** Graphical overview on processing of human tumor samples. After slicing or fragmentation of tumors, baseline samples were either fixed for histological evaluation or viability was determined by MTS assay. If the quality parameters were met, the sample was employed in permissivity analyses. Created in BioRender. Schoeps, B. (2025) https://BioRender.com/z20upzx. **B** Viability heterogeneity of tumor samples derived from 12 kidney cancer patients, each data point is derived from one tumor slice, *n* = 2–4 slices per patient. Bars indicate mean with standard deviation. Metabolic activity of tumor slices (**C**) or tumor fragments (**D**) derived from 8 different tumor entities. Each data point represents the mean corrected absorbance obtained from 1 to 6 slices or seeded fragment wells of one patient-derived tumor. Bars indicate mean with standard deviation. **E** Overview on the maximum corrected absorbance of tumor slices derived from 123 patient samples (1–6 slices per patient sample) and tested with the MTS assay. MTS values below the threshold obtained with Triton-X-treated slices (dead) were excluded from permissivity assessment. Bars indicate mean with standard deviation. Tumor content heterogeneity of tumor slices derived from an appendix tumor sample (**F**) and between different cancer entities (**G**); cholangiocellular cancer: *n* = 20 slices of 3 tumors, lung cancer: *n* = 18 slices of 3 tumors, kidney cancer: *n* = 29 slices of 3 tumors, pancreas cancer: *n* = 22 slices of 3 tumors, bladder cancer: *n* = 22 slices of 3 tumors. **H** Distribution of all tumor samples employed in this study with regard to exclusion criteria. **I** H&E pictures of representative baseline tumor samples employed in permissivity studies. Scale bar = 50 µm.

An important pre-requisite for permissivity testing of clinical samples is the quality of the acquired tumor samples. We therefore established a permissivity assay setup that includes a quality control of the tumor sample before cultivation (baseline) (Fig. 1A). This quality control includes metabolic measurements by MTS assay as well as histological evaluations of tumor cell content and histological integrity. We observed a strong heterogeneity in the metabolic activity of patient-derived tumor slices originating from the same tumor (Fig. 1B), as well as from different patients with the same tumor indication (Fig. 1B), and between different tumor indications (Fig. 1C). Interestingly, the heterogeneity in metabolic activity of different tumor regions was also obvious by visual inspection of tumor slices, where some areas showed strong MTS-mediated staining, while other areas were left unstained (Supplementary Fig. 1A). Of note, incubation of tumor slices with Triton-X abolished their metabolic activity (Supplementary Fig. 1B), indicating that the viability of tumor samples drives the MTS readout in our assay. Metabolic activity could also be determined in human tumor fragments (Fig. 1D). Employing MTS data of tumor slices that were pre-treated with Triton-X (dead slices), we retrospectively determined a viability threshold for the MTS assay, which was adjusted to exclude all tumors that showed metabolic activity below the Triton-X-treated slices (Fig. 1E and Supplementary Fig. 1B; threshold: corrected absorbance = 0.052). This was also performed for the tumor fragments approach (Supplementary Fig. 1C; threshold: corrected absorbance = 0.04).

Another key characteristic of the employed tissue is the tumor content, since absence of tumor cells in the sample could lead to false-negative permissivity data in the tested sample/tumor entity. Histological analyses of tumor tissues at baseline indeed revealed a very heterogenous pattern of tumor content, within tumor samples derived from the same patient (Fig. 1F), and also between different tumor entities (Fig. 1G). Overall, of the 179 tumor samples received, 21.7% were excluded after histological examination due to absence of cancer cells or high necrotic tissue content, and 3.9% due to low metabolic activity (Fig. 1H). In total, 133 samples (74.3%) of more than 20 different tumor entities were included to perform permissivity studies (Table 1 and Fig. 1I).

### Tracking OV replication in patient-derived tumor tissues

**Fig. 2:**
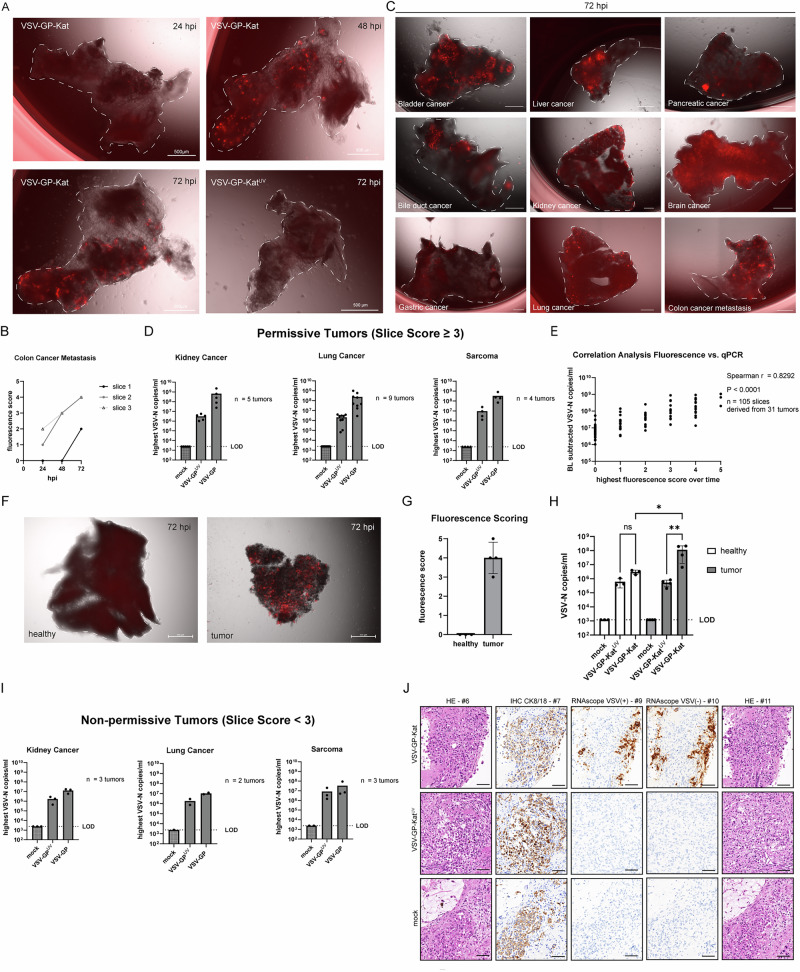
Tracking viral replication in patient-derived tumor tissues. Fluorescence microscopy pictures (**A**) and semi-quantitative scoring (**B**) of tumor slices derived from a metastasized colon cancer patient and infected with VSV-GP-Katushka (VSV-GP-Kat) or the UV-inactivated control (VSV-GP-Kat^UV^). **C** Representative fluorescence pictures of tumor slices derived from a variety of cancer indications (same tumors as shown in Fig. 1I) after infection with VSV-GP-Kat for 72 h. Scale bar = 500 µm. **D** qPCR analysis of supernatants (72 hpi) derived from VSV-GP-infected permissive tumor slices from kidney cancer (*n* = 5 tumors), lung cancer (*n* = 9 tumors) and sarcoma (*n* = 4 tumors). Maximum values obtained from 2 to 12 tumor slices per tumor are shown. Bars indicate means. LOD limit of detection. **E** Correlation between highest fluorescence score of tumor slices and VSV-N copies in the corresponding supernatant; *n* = 105 slices derived from 31 tumors. Spearman correlation analysis, *P* value < 0.0001. **F**–**H** Healthy liver tissue and liver tumor tissue were sliced and infected with VSV-GP-Kat. Viral replication was determined by fluorescence microscopy (**F**, **G**) and qPCR of the corresponding supernatant (**H**) at 72 hpi; tumor tissue: *n* = 4 samples per group, healthy tissue: *n* = 3 samples per group. Scale bar = 50 µm. Bars indicate means with standard deviation. Ordinary one-way ANOVA with Šídák’s multiple comparisons test; ns not significant, *P < 0.05, **P < 0.01. **I** qPCR analysis of supernatants (72 hpi) derived from VSV-GP-infected non-permissive tumor slices from kidney cancer (*n* = 3 tumors), lung cancer (*n* = 2 tumors) and sarcoma (*n* = 3 tumors). Bars indicate means. **J** RNAscope analysis of a colorectal cancer metastasis. Shown are sequential sections from a mock treated slice (bottom), from a slice treated with VSV-GP-Kat^UV^ (middle) and from a slice treated with VSV-GP-Kat (top). Sections are stained with HE (sections #6 and #11), immunohistochemistry for anti-CK8/18 (section #7, Leica #5D3-L-CE, HIER1, 1:250, IHC_F) as well as RNAscope for VSV-(+)-strand (section #9) and VSV-(−)-strand (section #10). Scale bar = 50 µm.

To determine permissivity of human tumor tissues to OVs, we first monitored viral replication in the well-established tumor slice culture system [17]. Real-time fluorescence microscopy revealed increasing transgene expression of VSV-GP-Katushka (VSV-GP-Kat) over time (Fig. 2A, B). Importantly, viral replication and the associated Katushka expression could be observed in tumor entities derived from various tissues, for example bladder, liver, pancreas, bile duct, kidney, brain, gastric, lung and colon cancer (Fig. 2C), indicating that the established setup works independent of the tumor origin. Of note, histological evaluation also revealed that most tumor samples retained tissue integrity and tumor content during cultivation for 72 h (Supplementary Fig. 2A). Next, we aimed to simplify the readout for permissivity. We chose qPCR as reproducible readout of viral replication since it can easily be run in a great number of laboratories and is widely used as readout of viral replication [22]. Because lysing tumor tissues after cultivation would impede subsequent histological analyses, we determined viral genome copies as readout of viral replication in the supernatant of tumor samples and used the corresponding tissue for histological analyses. To differentiate between input viral genomes and active viral replication, we included an UV-inactivated VSV-GP control (VSV-GP-Kat^UV^) [17, 20]. Indeed, we observed an increase in VSV-N copies, as compared to the UV-inactivated virus control, in the supernatant of permissive tumor samples derived from various cancer entities, for example kidney cancer, lung cancer, and sarcoma (Fig. 2D). The values ranged between 2.88E + 06 and 2.32E + 09 VSV-N copies/ml, depending on the tested tumor entity (Fig. 2D). Evaluating tumor samples derived from 31 cancer patients, we also observed a significant correlation between the microscopically determined fluorescence score and VSV-N copies of the corresponding tumor, indicating that both approaches provide comparable readouts of permissivity (Fig. 2E).

To determine the tumor specificity of VSV-GP replication, we compared permissivity of healthy liver tissue with permissivity of liver metastases of the same patient. Importantly, no replication could be detected in the healthy liver control, whereas strong replication was obvious in the sample exhibiting a liver metastasis (Fig. 2F–H). While we saw a strong replication in most tumor tissues, we also observed that several patient-derived tumor samples were non-permissive (Fig. 2I). Of note, differences in VSV-N copies of less than 10-fold compared to the UV-inactivated control were interpreted as no active viral replication, since UV-inactivation itself slightly reduced the amount of detectable VSV-N copies (Supplementary Fig. 2B), similar to recent observations [20].

Since the assay setup involves sample fixation and histological analyses at the endpoint (i.e. at 72 h post infection (hpi)), the obtained FFPE samples could also be used for the detection of viral replication in situ. We therefore performed RNAscope, which can be used to detect replication of VSV-GP by differentiating between positive- and negative-strand RNA [20]. Indeed, RNAscope further confirmed viral replication in permissive cultivated tumor samples (Fig. 2J).

### Systematic comparison of human tumor models for permissivity to VSV-GP

**Fig. 3:**
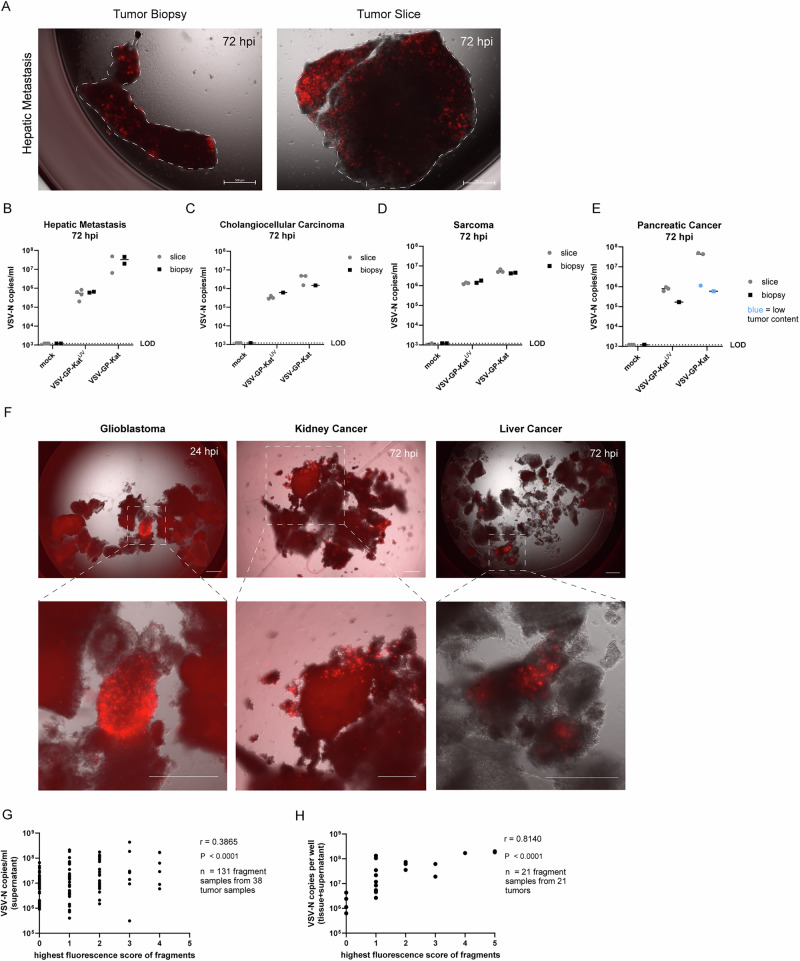
Systematic comparison of human tumor models for permissivity to VSV-GP. **A** Representative fluorescence microscopy pictures of a tumor biopsy and a tumor slice derived from the same hepatic metastasis and infected with VSV-GP-Kat at 72 hpi. Scale bar = 500 µm. **B**–**E** VSV-N copies in supernatants derived from tumor biopsies and tumor slices of the same patient sample. Each data point represents data obtained from one tumor slice or one tumor biopsy; **B** hepatic metastasis, **C** cholangiocellular carcinoma, **D** sarcoma, **E** pancreatic cancer. Blue data points indicate tumor samples with low tumor content. Bars indicate the median. **F** Representative fluorescence microscopy pictures of tumor fragments infected with VSV-GP-Kat for the indicated times. Scale bar = 500 µm. Correlation between fluorescence score of tumor fragments and VSV-N copies in the corresponding supernatant (**G**, *n* = 131 tumor fragment samples derived from 38 tumors) or whole-well lysates (**H**, *n* = 21 tumor fragments obtained from 21 tumors). Spearman correlation analysis, *P* value < 0.0001.

Next, we aimed to simplify this permissivity assay and identify the optimal human tumor model for permissivity testing. Such a tumor model should combine parameters of practicality and clinical translatability. On the one hand, it should allow for rapid and simple handling, require no specialized equipment or training, and offer high reproducibility. On the other hand, it should accurately reflect the patient’s tumor, including preservation of the native structure and complexity of the TME. First, we compared patient-derived tumor slices versus tumor punch biopsies which both were obtained from the same surgically removed hepatic metastasis sample. Indeed, replication of VSV-GP-Kat in tumor biopsies could be detected both by fluorescence microscopy as well as by qPCR, similar to the established tumor slice model (Fig. 3A, B). Both approaches also provided similar results for non-permissive tumor samples from an intrahepatic cholangiocellular carcinoma (Fig. 3C), and a sarcoma (Fig. 3D). However, we also observed differences between both approaches, e.g. tumor samples derived from a pancreatic cancer patient were found to be permissive in the slice approach but not in the biopsy setting (Fig. 3E). This difference was caused by sampling issues, since we observed that the tumor content was high in most of the obtained tumor slices, but low in the obtained tumor biopsy (Fig. 3E, blue data points).

Given that this large heterogeneity compromised a direct comparison of the two approaches, we decided to run a direct comparison between tumor biopsies and slices in the more controlled and homogenous setting of subcutaneous murine tumors. Infection of ex vivo biopsies and slices of highly permissive CT26-CL25-IFNAR^−/−^ murine colon tumors revealed that replication was similar in both approaches (Supplementary Fig. 3A). Of note, we also observed that the inter-tumor and intra-tumor variability of the murine biopsy approach was very low (Supplementary Fig. 3B) and that OV replication in the supernatant of murine tumor biopsies was comparable to the results obtained in tissue lysates (Supplementary Fig. 3C).

Although the acquired results support the use of tumor biopsies as simple alternative for tumor slices, we noticed that the tissue properties of a variety of surgically removed tumor samples precluded the collection of punch biopsies. These properties encompass tissues characterized by soft and compressible consistency, high rigidity or fibrous composition. The punch biopsy approach might therefore be limited to tumors with appropriate tissue properties or tumor biopsies that are directly obtained from the clinics.

In the next step we therefore tested tumor fragments as an alternative approach for permissivity testing. Since the fragments are generated by cutting whole tumor specimens into small pieces, the chance of sampling errors is reduced. In addition, the obtained fragment suspension can be easily split up into several wells to run the assay in replicates. Indeed, replication of VSV-GP was observed by fluorescence microscopy in tumor fragments derived from several tumor entities, for example glioblastoma, kidney cancer, and liver cancer (Fig. 3F). Since we only observed a weak correlation between fluorescence of tumor fragments and VSV-N copies in the corresponding supernatants (Fig. 3G), we determined VSV-N copies in the whole well, including supernatant as well as the corresponding tissue lysate. This setup showed a strong correlation between fluorescence and viral genomes (Fig. 3H), indicating that the release of viral genomes to the supernatant might be compromised in the fragment approach. In sum, the tumor fragment approach was identified as a simple and very well manageable alternative to tumor slices.

### An assay for semi-automated permissivity testing

Although the obtained results indicate that qPCR is a suitable approach for a permissivity assay, it lacks the possibility of real-time tracking of viral replication, is time consuming, costly, labor-intensive, and shows substantial background due to the input virus. To overcome these limitations, we set out to find an alternative readout for viral replication that can be employed in the permissivity assay. For this, we engineered VSV-GP to express the NanoLuc luciferase, which shows a > 150-fold higher sensitivity than firefly or *Renilla* luciferases and has a size of only 19.1 kDa [23, 24], allowing incorporation into viruses with small transgene capacity. Furthermore, the available substrate Endurazine (Promega Corporation) for NanoLuc is stable for up to 72 h in culture media and is only metabolized by living cells to the active substrate furimazine. This setup allows nonlytic real-time expression analyses for periods lasting several days, which makes it an ideal system for permissivity studies. To compare the luminescence readout with fluorescence microscopy and qPCR analyses, we linked expression of NanoLuc to Katushka, allowing simultaneous tracking of viral replication by luminescence and fluorescence.

**Fig. 4:**
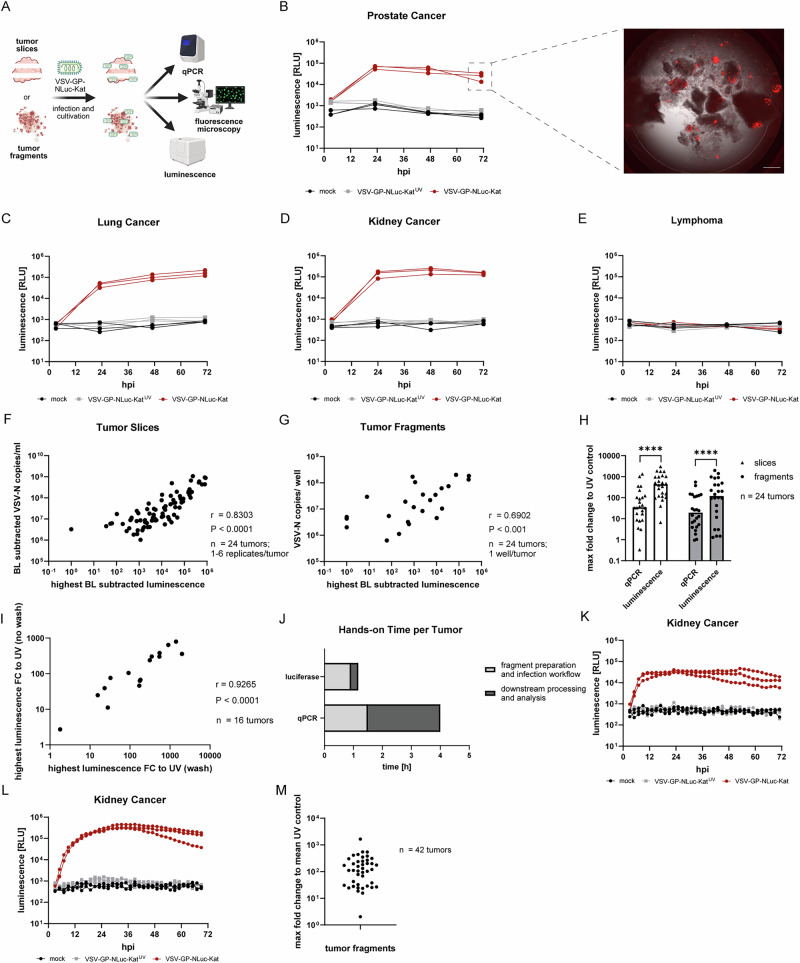
An assay for semi-automated permissivity testing. **A** Graphical overview on permissivity testing of human tumor slices and fragments. After infection with VSV-GP-NLuc-Kat, tumor samples are cultivated for 72 h and permissivity is determined by qPCR, fluorescence microscopy, and luminescence. Created in BioRender. Schoeps, B. (2025) https://BioRender.com/xy0gjuk. Representative examples of tumor fragments derived from a prostate cancer patient (**B**), a lung cancer patient (**C**), a kidney cancer patient (**D**), and a lymphoma patient (**E**). The luminescence signal of tumor fragments infected with VSV-GP-Nluc-Kat was monitored for 72 h. A representative fluorescence microscopy picture was taken 72 hpi (**B**). Scale bar = 500 µm. Correlation between luminescence of tumor slices (**F**) or tumor fragments (**G**) and VSV-N copies in the corresponding supernatant (**F**) or whole well (**G**). *n* = 81 slices derived from 24 tumors and *n* = 24 tumor fragment samples derived from the same tumors; Spearman correlation analysis. **H** Comparison of qPCR and luminescence as readout of viral replication in tumor fragments and tumor slices derived from 24 tumors. Indicated as fold change to the UV-inactivated VSV-GP-Nluc-Kat control. Bars indicate the median. Wilcoxon test, ****P < 0.0001. **I** Correlation between luminescence of tumor fragments derived from 16 tumors that were washed at 2 hpi and tumor fragments of the same tumors that were not washed after infection with VSV-GP-Nluc-Kat. Spearman correlation analysis, *P* value < 0.0001. **J** Comparison of hands-on-time between the luminescence approach and qPCR. Fragment preparation and infection workflow: fragment generation, infection, MTS assay, wash (qPCR), Endurazine addition (luminescence), collection of supernatant (qPCR), fixation of samples; downstream processing and analysis: documentation, RNA extraction (qPCR), run qPCR, analysis. **K**, **L** Semi-automated real-time tracking of viral replication employing the BioSpa-Synergy H1 combination. Tumor fragments of kidney cancer patients were infected with VSV-GP-Nluc-Kat or the UV-inactivated control (VSV-GP-Nluc-Kat^UV^) and monitored for 72 hpi in 2 h intervals. Each data point represents one tumor fragment well. **M** Maximal fold change to the UV-inactivated VSV-GP-Nluc-Kat control that was obtained with the tumor fragment approach in each tumor. *n* = 42 tumors.

To employ the NanoLuc approach for permissivity testing, we first tested the impact of the substrate Endurazine on viability of human tumor fragments. Importantly, we did not observe an effect of Endurazine on the metabolic activity of human tumor fragments after 48 h of cultivation (Supplementary Fig. 4A), indicating that the substrate does not impair tissue viability. Thus, we proceeded with infection of tumor fragments and tumor slices derived from the very same patient sample with VSV-GP expressing the new NanoLuc-Katushka marker gene combination (VSV-GP-NLuc-Kat) (Fig. 4A). Indeed, viral replication could be tracked in real-time by detection of luminescence, as well as fluorescence microscopy of tumor fragments (Fig. 4B–E). Importantly, detection of luminescence allowed kinetic analyses of viral replication in tumors of several entities, including prostate cancer (Fig. 4B), lung cancer (Fig. 4C) and kidney cancer (Fig. 4D). Of note, also non-permissive tumors were identified, including tumor fragments derived from a lymphoma patient (Fig. 4E). The luminescence approach also showed a strong correlation with viral replication determined by qPCR in tumor slices (Fig. 4F) as well as fragments (Fig. 4G) derived from a total of 24 different tumors, including lung cancer, gastric cancer, bladder cancer, melanoma, colorectal cancer, kidney cancer and pancreatic cancer. To define an appropriate background control for the luminescence readout, we evaluated several conditions. We found no measurable differences in luminescence between substrate incubated with mock‑infected tumor slices, slices infected with VSV‑GP‑NLuc‑Kat^UV^, or samples containing substrate with only input VSV‑GP‑NLuc‑Kat^UV^ or input VSV‑GP‑NLuc‑Kat at either 48 or 72 hpi (Supplementary Fig. 4B, C). Likewise, the background fluorescence detected in substrate incubated with culture medium alone was comparable to that observed in tumor slices infected with VSV‑GP‑NLuc‑Kat^UV^ (Supplementary Fig. 4D, E). This indicates that it is sufficient to run VSV-GP-NLuc-Kat^UV^–infected samples as background controls for the luminescence assay. To compare the permissivity of different tumors and approaches (qPCR vs. luminescence) we decided to normalize the obtained luminescence data to the respective VSV-GP-NLuc-Kat^UV^ control. The resulting fold change to the UV control serves as indicator of viral replication in each sample. This analysis revealed a higher fold change in the luminescence approach as compared to qPCR (Fig. 4H), indicating a higher signal to noise ratio in the luminescence approach. To also reduce the hands-on time of the assay, we removed the wash step after virus infection, which was only introduced to remove the input virus for subsequent qPCR. Of note, we observed a strong correlation between luminescence of washed and non-washed tumor fragments of the same tumor sample, indicating that this procedural change had no effect on the readout of the assay (Fig. 4I). In sum, the luminescence approach reduced the hands-on time of the assay from ~4 h to just over 1 h per tumor sample (Fig. 4J).

In a final attempt to further improve the usability of the assay by minimizing manual intervention and also gain more information on the kinetics of viral replication, we tested whether the established setup can be run in a semi-automated manner. For this, VSV-GP-NLuc-Kat-infected tumor samples were incubated in a BioSpa-SynergyH1 (Agilent) incubator-reader-combination for 72 h with automated detection of luminescence every 2 h. This setup provided so-far unprecedented details in virus replication that have not been achieved by the other tested methods. For example, we observed a peak in viral replication already at 12 hpi in a tested tumor sample (Fig. 4K), whereas slower virus replication was observed in another sample (Fig. 4L). Due to these differences in kinetics of viral replication, we focused on the highest value that was obtained during the 72 h of cultivation in order to determine permissivity. This analysis revealed that the majority of tested tumors showed high virus replication (Fig. 4M).

### Transfer of the permissivity assay to an independent laboratory

**Fig. 5:**
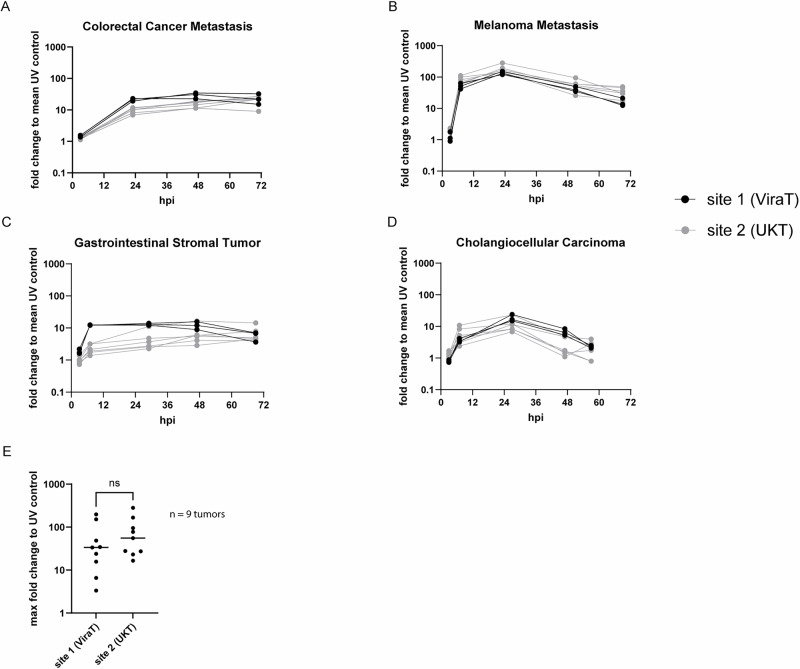
Transfer of the permissivity assay to an independent laboratory. **A** Real-time tracking of viral replication at two independent laboratories. Tumor fragments derived from a colorectal cancer metastasis (**A**), a melanoma metastasis (**B**), a gastrointestinal stromal tumor (**C**), and a cholangiocellular carcinoma (**D**) were infected with VSV-GP-Nluc-Kat or the UV-inactivated control (VSV-GP-Nluc-Kat^UV^) and luminescence was monitored for up to 72 hpi. Each data point represents one fragment well; *n* = 3–6 fragment wells per tumor. **E** Comparison of the maximal detected fold change in luminescence for each tumor; *n* = 9 tumors. Bar indicates the median. Wilcoxon test; ns not significant.

To verify robustness of the established permissivity assay, we transferred it to an independent laboratory and compared permissivity of the same patient-derived tumor samples at both sites in parallel. For this, surgically removed tumor tissues were split into two representative pieces, which were then transported overnight to the respective labs to run the permissivity assay (tumor fragments, VSV-GP-NLuc-Kat, luminescence approach). Since the automated incubator was not available at both sites, the experimental setup was simplified to a 24 h interval of luminescence detection. This analysis revealed similar permissivity results obtained by both laboratories in a variety of tumor samples, including colorectal cancer metastasis (Fig. 5A), melanoma metastasis (Fig. 5B), gastrointestinal stromal tumor (Fig. 5C), and cholangiocellular carcinoma (Fig. 5D). Although slight differences in the kinetics of viral replication could be observed in some tumors (Fig. 5C), no significant difference was observed in the maximal luminescence fold change to the UV-inactivated control within 72 hpi (Fig. 5E). These results clearly demonstrate that the established permissivity assay, i.e. luminescence detection in VSV-GP-NLuc-Kat-infected tumor fragments, generates robust and reproducible data independent of the laboratory or testing location and can be easily and successfully implemented at different sites.

## Discussion

Although the development of immunotherapies has revolutionized oncology, the majority is still approved without identification of the ideal target population [25], and only 5.3% of cancer drugs that enter phase I clinical trials finally obtain approval [26]. This gap in clinical translation is particularly evident in OV therapy, where so-far no biomarkers for clinical benefit have been identified [1]. Furthermore, the currently employed murine models have several important limitations that impair the translation of preclinical findings [1, 14]. Thus, model systems that better capture the complexity of the tumor heterogeneity seen in patients [27], for example patient-derived tumor models, also termed patient tumor avatars [15, 16], should be employed especially in the context of biologically complex oncolytic virotherapy [28]. Despite the existence of several human tumor models and their application in OV studies [13, 17, 18], a systematic approach to develop a permissivity assay has not been undertaken yet.

Here, we established an easy-to-use reproducible permissivity assay that allows real-time tracking of OV replication in patient-derived tumor samples. To evaluate the suitability of different ex vivo tumor models for the permissivity assay, we compared three model systems - tumor slices [17, 29], tumor biopsies [18, 30] and tumor fragments [19] - all of which preserve the TME, a critical factor known to control the replication of OVs in tumors [10]. The impact of the TME on replication of OVs in cancer cells is determined on the one hand by cellular factors like stromal and immune cells but also by acellular factors like nutrient availability [10]. Thus, it might be compelling to link the stromal content of employed tumor samples to their permissivity. Given that tissue digestion and dissociation of tumors disrupts the patient-specific native 3D microenvironment, we excluded the widely applied organoid model from our comparison. Although the tested ex vivo tumor models have previously been utilized to assess responses to various therapeutic agents - including studies on OV replication in tumor biopsies and slices [17, 18] - comparative data on OV replication across these models were lacking and it was unclear which of these might be most suitable for a broadly-applicable permissivity assay.

Intra‑tumoral heterogeneity represents an important factor influencing permissivity measurements in ex vivo assays. In our study, variability observed between replicate tumor slices or fragments derived from the same patient sample likely reflects differences in tumor cell content rather than procedural inconsistencies. This is exemplified in Fig. 3E, where slices with high tumor content from a pancreatic cancer specimen supported robust viral replication, whereas slices with low tumor content from the same lesion did not. Such variability is inherent to primary human tumor material and depends strongly on the specific region of the tumor that is sampled. Consistent with this interpretation, we observed substantially lower variability in murine subcutaneous tumors - which are more homogeneous by nature - compared with human samples (Fig. 2D vs. Supplementary Fig. 3). Rather than undermining the robustness of the assay, these findings underscore the importance of appropriate sampling strategies and the use of biological replicates to adequately capture intrinsic tumor heterogeneity. In addition to heterogeneity of permissivity in general we also observed variations in the kinetics of viral replication. In patient-derived tumor tissues, reporter signals can peak as early as 12 h post infection, whereas other samples showed much slower replication of VSV-GP. While an early positive signal allows rapid identification of permissive tumors, the full 3-day assay window is required to capture also slower replicating samples and minimize false negative results. From a clinical perspective, a turn-around time span of 5–7 days, which is needed to obtain the results of permissivity assays, would not delay the onset of virotherapy, neither when performed within the screening period of clinical studies, nor in the context of routine applications of licensed virotherapeutic compounds. Altogether, our data highlight the importance of considering tumor heterogeneity in the development of new OV therapies.

Even though we observed similar levels of viral replication in the tested models, we decided to focus on tumor fragments in the final assay setup because of the following advantages of this approach. First, tumor fragments are easy to obtain and do not need special training or equipment compared to the slice approach. Second, they can be generated from virtually all tumor samples, independent of the tissue characteristics. This contrasts with the biopsy approach where we found, unlike previous observations [18], that obtaining punch biopsies from soft and compressible tumors, as well as samples with high rigidity or fibrous composition was technically not feasible.

In the next step towards a straightforward permissivity assay, we compared three different approaches to monitor viral replication, namely fluorescence microscopy, qPCR and luminescence. Although viral replication was evident in all three approaches, luminescence appeared to be superior for several reasons. First, the approach is straightforward and can be run in a semi-automated manner. This not only reduced the hands-on-time of the assay but also allowed a very close monitoring of viral replication, leading to detection of differences in viral replication kinetics between tumors that would have remained undetected using alternative approaches. In addition, the signal-to-noise ratio in the luminescence approach is higher as compared to qPCR and fluorescence microscopy. One reason for this might be the high background signal produced by the input virus in the qPCR setup. The observation that tumors show substantial levels of autofluorescence depending on the tumor differentiation [31] might be one reason why we could not detect expression of reporter proteins by fluorescence microscopy in some tumor slices (low fluorescence score) even though the VSV-N copies in the corresponding supernatant indicated active viral replication (Fig. 2E). During assay development, we also found that HEK293 cells provided a robust and highly consistent positive control for detecting viral replication (data not shown), and they could be used as an internal standard by laboratories that routinely maintain cell cultures. However, because the assay was designed for routine use in a clinical setting, where integrating cell‑line–based controls would add logistical complexity, we chose not to incorporate a cell‑based control into the final assay design.

While this assay was established by employing the OV VSV-GP-NLuc-Kat, the NanoLuc enzyme can easily be expressed also by other OV platforms and the assay is thus applicable to a wide range of viruses. However, several aspects need to be considered in case other OVs will be used. First, it is possible that the threshold of luminescence signal for determination of tumor permissivity varies between OV platforms and should thus be optimized to the OV employed. Second, the replication kinetics of various OV platforms differ [32], which could be an issue in case the viability of tumor tissues is compromised during extended cultivation. For example, it has been shown that cultivation of tumor slices for 6 days reduces tissue viability and requires optimization of the cultivation method [29]. Since this assay was established to work with a variety of tumor indications, we did not optimize the culture conditions for long-term cultivation of specific entities. This might be necessary if cultivation for more than 3 days is envisioned. In addition to tissue viability, extended cultivation periods could also impair substrate stability, and it might be necessary to readminister the substrate Endurazine at later timepoints. The stability of the NanoLuc enzyme is likely not an issue, since it is quite stable even at decreasing pH [24]. Finally, prolonged ex vivo cultivation of tumor samples might also lead to molecular and cellular changes that negatively impact the translatability of the obtained results.

Human tumor models play a pivotal role in advancing various OV concepts, as they overcome key limitations of murine models, including differences in viral cell tropism and tumor heterogeneity [1, 14]. However, they lack the systemic immune response characteristic of complex in vivo systems. Given that the therapeutic efficacy of OVs is mediated not only by direct lysis of permissive tumor cells, but also by the induction of anti-tumor immunity through replication-independent mechanisms [33–35], permissivity alone may not be sufficient to predict clinical outcomes. For example, it has been shown that VSV elicits tumor regression and durable cures also in infection-resistant tumor models via systemic activation of anticancer immunity [34]. Interestingly, it has also been shown that single-cycle viral gene expression, rather than progressive replication and oncolysis, is required for VSV therapy of B16 melanoma [33]. Therefore, it is important to distinguish tumor permissivity from productive infection. In this work, we define permissivity as the ability of tumor tissue to support viral genome replication and protein expression, which we quantify with the described assay. Although we do not directly measure the production of infectious progeny, the increasing luminescence and fluorescence signals suggest ongoing viral gene expression and likely secondary infection events. Because viral replication itself can drive downstream immunogenic effects, and viral gene expression has been found to be strongly correlated with in vivo therapy response [33], measuring replication remains a meaningful readout even without formal assessment of viral productivity. The efficacy of OV therapy is thus driven by two central pillars, tumor permissivity and the antiviral immune response, which can be further potentiated by replicating OVs [10]. Studies correlating ex vivo permissivity of human tumors with virotherapeutic efficacy in corresponding cancer patients are therefore essential to establish the clinical relevance of the described permissivity assay. In the absence of predictive biomarkers for OV activity [1], such studies would represent a critical step towards improving the clinical success rate of OV trials. In addition, we acknowledge that the number of samples per tumor entity in this study is limited, which does not allow for drawing generalizable conclusions about entity specific permissivity patterns. The inherent heterogeneity and potential intra-entity variability in permissivity require substantially larger patient cohorts to reliably characterize permissivity profiles of individual tumor types. Here, we established a robust ex vivo assay that can serve as a methodological foundation for such larger-scale investigations. Future studies employing expanded patient cohorts for individual tumor types will be essential to translate this assay into an entity-focused biomarker discovery tool. Ultimately, this assay may serve as valuable pre-clinical tool to aid appropriate patient selection and prioritize tumor indications in future OV clinical trials.

## Supplementary information

Supplementary Figures

## Data Availability

All data are available within the article and its supplemental information.
