# Supplementary material for: An ex vivo permissivity assay to assess replication of the oncolytic virus VSV-GP in patient-derived tumor samples
Source: Oncogene. 2026 May 26;45(26):2552–66. doi: 10.1038/s41388-026-03822-9 (PMC13286994; doi:10.1038/s41388-026-03822-9)
Supplement: Supplementary file 1 — Supplementary Figures [file 41388_2026_3822_MOESM1_ESM.pdf]

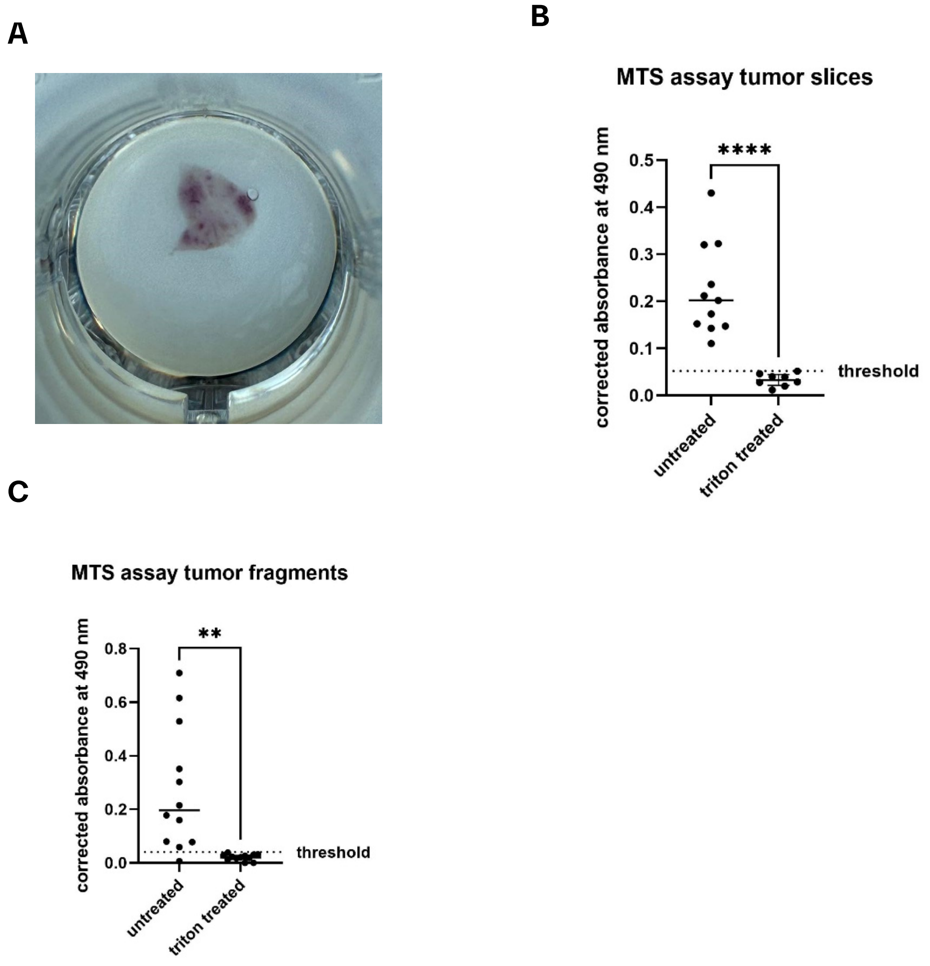

Supplementary Figure 1: Viability assessment of human tumor samples. (A) Representative picture of a tumor slice derived from a prostate cancer sample after viability assessment via MTS assay. The dark areas indicate tumor areas with high metabolic activity. (B+C) Determination of background MTS assay absorbance in Triton-X-treated (dead) tumor slices (B, n = 2-6 tumor slices derived from 3 patient-derived tumors) or tumor fragments (C, n = 3 fragment wells of 4 tumors). Unpaired t test, ** P < 0.01, **** P < 0.0001; line indicates median.

A

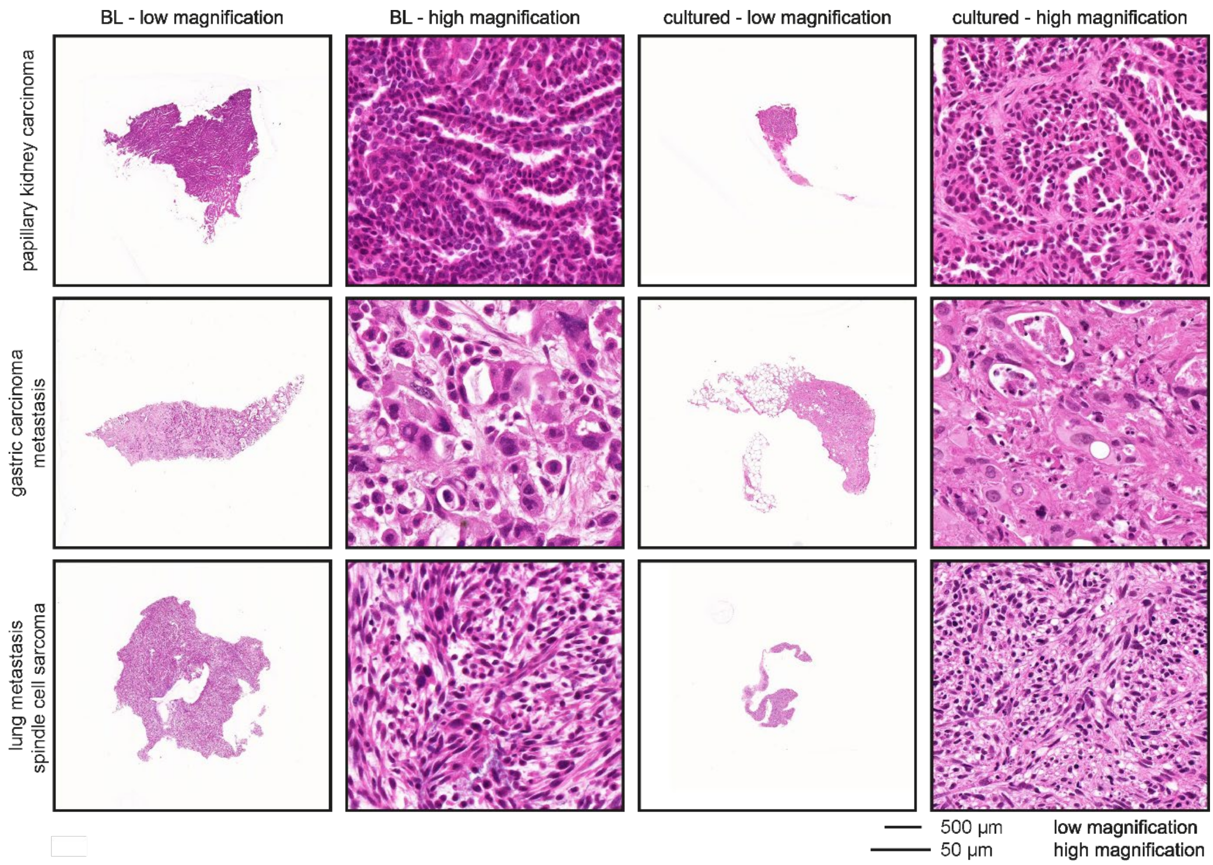

B

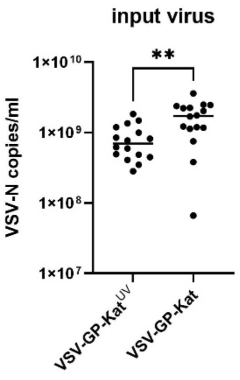

Supplementary Figure 2: Permissivity testing of human tumor samples – controls. (A) Histological evaluation of tumor slices at baseline and 72 hpi. (B) UV-inactivation of VSV-GP-variants slightly reduces VSV-N copies. n = 16 samples; line indicates median, Mann Whitney test, ** $P < 0.01$.

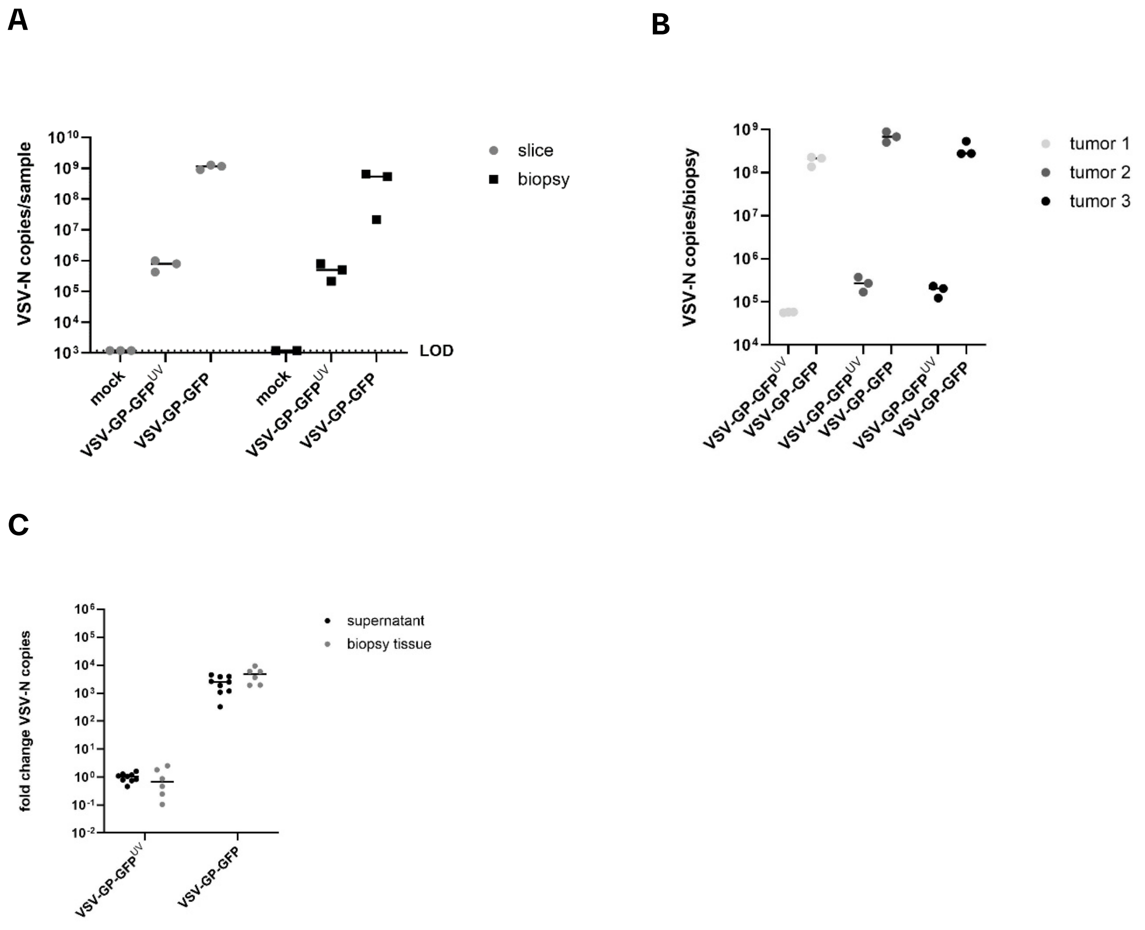

Supplementary Figure 3: Replication of VSV-GP in murine tumor biopsies. (A) VSV-N copies in lysates of tumor slices or biopsies derived from a murine CT26-CL25-IFNAR$^{-/-}$ tumor after infection with VSV-GP-GFP for 17 h. Each dot represents the data obtained from one tumor slice/biopsy. Bars indicate the median. (B) qPCR analysis of lysates derived from CT26-CL25-IFNAR$^{-/-}$ murine tumor biopsies infected with VSV-GP-GFP or the UV-inactivated control (VSV-GP-GFP^{UV}) for 17h. n = 3 tumor biopsies derived from 3 murine tumors. (C) Biopsies derived from CT26-CL25-IFNAR$^{-/-}$ murine tumors were infected with VSV-GP or the UV-inactivated control (VSV-GP-GFP^{UV}) for 12h, then washed once with media. Supernatants and biopsy tissues were collected 48 hpi and VSV-N copies were analysed via qPCR. n = 6-9 biopsies derived from 4 tumors.

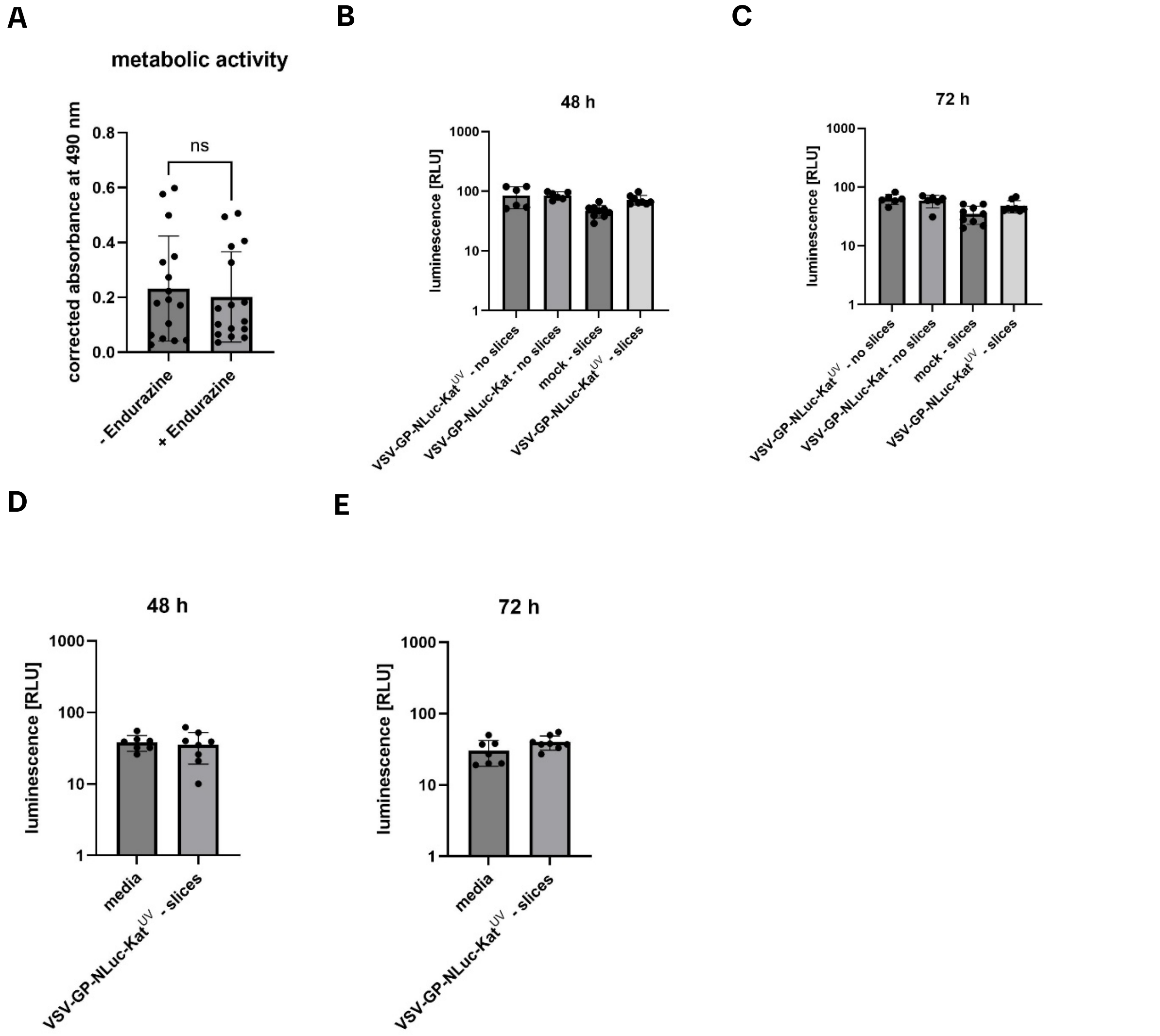

Supplementary Figure 4: NanoLuc as permissivity readout. (A) Human tumor fragments were incubated with Endurazine for 48h and viability was assessed by MTS assay. n = 16 fragment wells of 4 tumors; mean with SD; Mann Whitney test. (B+C) Luminescence detection of input virus (VSV-GP-NLuc-Kat^{UV}, VSV-GP-NLuc-Kat), mock infected human tumor slices and VSV-GP-NLuc-Kat^{UV} infected human tumor slices 48 h after cultivation (B) or 72 h after cultivation (C). Mean with SD; n = 6-9 samples each. (D+E) Luminescence detection of media and VSV-GP-NLuc-Kat^{UV} infected human tumor slices 48 h after cultivation (D) or 72 h after cultivation (E). Mean with SD; mock: n = 7; VSV-GP-NLuc-Kat^{UV}: n = 8.
